# Efficient Lévy walks in virtual human foraging

**DOI:** 10.1038/s41598-021-84542-w

**Published:** 2021-03-04

**Authors:** Ketika Garg, Christopher T Kello

**Affiliations:** Department of Cognitive and Information Sciences, University of California, Merced, CA 95343 USA

**Keywords:** Behavioural ecology, Human behaviour

## Abstract

Efficient foraging depends on decisions that account for the costs and benefits of various activities like movement, perception, and planning. We conducted a virtual foraging experiment set in the foothills of the Himalayas to examine how time and energy are expended to forage efficiently, and how foraging changes when constrained to a home range. Two hundred players foraged the human-scale landscape with simulated energy expenditure in search of naturally distributed resources. Results showed that efficient foragers produced periods of locomotion interleaved with perception and planning that approached theoretical expectations for Lévy walks, regardless of the home-range constraint. Despite this constancy, efficient home-range foraging trajectories were less diffusive by virtue of restricting locomotive search and spending more time instead scanning the environment to plan movement and detect far-away resources. Altogether, results demonstrate that humans can forage efficiently by arranging and adjusting Lévy-distributed search activities in response to environmental and task constraints.

## Introduction

Human intelligence appears to have an evolutionary basis, at least in part, in foraging and other search activities of our hunter-gatherer ancestors^1,2^. Presumably, survival depended not only on our ability to search effectively but also to flexibly adapt to goals and constraints as they arise and change. Goals and constraints are influenced by environmental conditions–such as terrain features, energy expenditure, and distribution of food and other resources–and by individual and social factors, such as the need to return to home and participate in social activities^3^. Efficient foragers need to manage the trade-offs that these conditions and factors are likely to present. Our ancestors may have used and honed their cognitive capacities for managing trade-offs and other foraging functions. However, less sophisticated strategies can be effectively deployed when knowledge or cognitive capacities are lacking. In particular, random search patterns can be efficient without requiring much memory, planning, or decision-making.

**Figure 1:**
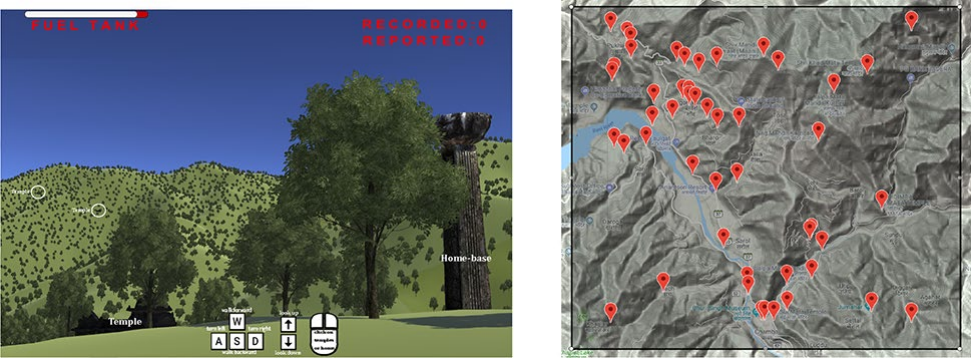
Game Environment. Left: An illustrative view of the virtual foraging game (key and mouse icons not shown during game play). Keys were used to move forward or backward (‘w’ or ‘s’), turn left or right (‘a’ or ‘d’), or tilt the view (‘up’ or ‘down’). The mouse was used to click on temples or the home-base. The ‘fuel tank’ showed the current energy remaining, ‘reported’ showed a running count of temples scored, and ‘recorded’ showed the current number of temples found but not yet reported to home-base (home-range condition only). Right: Distribution of selected temples in the Google Maps (Maps data ©2018) view of the Himalayan terrain.

In random search, movement displacements and pauses between movements can vary stochastically to effectively explore the environment^4–7^. Efficient random search has been formalized in terms of *Lévy walks* in which displacement sizes are drawn from a heavy-tailed distribution, and the probability of observing a given displacement is inversely proportional to its length(*l*), $P(l) \sim l^{-\mu }$; $1 < \mu \le 3$. The Lévy exponent $\mu = 2$ generates superdiffusive search trajectories when directions are chosen at random at every step. Lévy walks generate displacements over a large range of scales that allow efficient sampling of an uncertain environment^8^. Such trajectories can balance extensive exploration of the search space that reduces the chances of revisiting areas with intensive coverage of immediate surroundings, as is beneficial when resources are scarce and their locations unknown. Studies have reported evidence for Lévy-like foraging in many different species, including humans^9^. For example, Lévy walks were observed in hunter-gatherer tribes in the Dobe Ju/’Ohoansi population^10^, and the Hadza of Tanzania^11^. The pauses between movements can have effects on movement patterns^12–14^, and they can be essential for resting, resource detection, and visual scanning. The lengths of pause durations can also be distributed akin to Lévy walks^15–17^, which we return to later.

Lévy walks increase search efficiency under the assumption of perfect, effortless detection of sparse resources. However, real perceptual systems are less than perfect and perceptual accuracy requires effort and concentration. For instance, the proportion of time and effort spent on relatively intensive search activities can increase with the difficulty of resource detection^18–20^. Furthermore, the difficulty of resource detection can increase with movement speed^21^ as a form of speed/accuracy trade-off^22,23^. More generally, attention is a limited resource and must be selectively allocated based on prevalent foraging conditions^24–26^. Thus, the flexibility in search strategies necessary to adapt to diverse environments might stem from the combination of locomotive and perceptual processes^27^, and a full understanding of foraging behavior requires an integration of the two. Despite the interdependence of locomotion and perception, they need not be affected by similar constraints. For instance, locomotion is inherently constrained by the landscape and energy expenditure^28,29^, whereas perceptual accuracy is constrained by time and ability to concentrate^20^.

The roles of perception and attention highlight how foraging is a *multiscale* process–relatively local perceptual and attentional processes unfold on shorter timescales, and these processes must be coordinated with movement and decision-making processes that unfold over a broader range of temporal and spatial scales^30,31^. Previous studies illustrate how foraging decisions in humans and other complex organisms are guided to minimize time and energy expenditure. However, decision-making is itself a process that primarily expends time and effort, and energy to a lesser degree^32^. Despite this rationale, there is little research on how time and energy are managed to make foraging decisions and movements. This research question is challenging to address in natural foraging because of the lack of experimental control and inability to measure behavior at a resolution needed for data on perceptual and decision-making processes. Some studies of human foraging have used simplified tasks and games to run controlled experiments^33–35^, but these studies mostly lack essential features like energy costs^36,37^ and multiscale interactions between perception, movement, and decision-making that are the bases of efficient foraging.

## Experiment

We designed a virtual environment to study human foraging in a natural setting that engages multiscale processing while also affording experimental control and a range of detailed measurements (links to the game can be found in Methods). We aimed to investigate the extent to which efficient human foraging is founded on basic search processes and how search processes are augmented by decision-making based on trade-offs between effort expended on movement versus planning and perception. The primary trade-off occurs between local versus non-local search processes, i.e., those driven by sensory activity versus locomotive activity, akin to the trade-off between intra-patch versus inter-patch foraging^3,38^. The present study examines how this trade-off is managed for efficient foraging under different task conditions.

We replicated a region in the foothills of the Himalayas using Google Maps and the Unity 3D game engine (Fig. 1). The region has several ancient temples whose locations we used to model the distribution of resources to be foraged for. These temples are spatially distributed according to human on-foot movement patterns and various socio-cultural and environmental factors^39^. Historically, they may have served as waypoints for journeys of nomadic tribes of the region^40^. The region is heterogeneous in terms of terrain elevation and visibility, thereby invoking naturally complex decision-making about energy expenditure for locomotion^28^ aimed at exploration versus reaching better vantage points for visual search^32^. Players foraged by moving through the environment at a constant human-scale velocity, and energy was expended as an empirically-based function of change in elevation^41^ (See Methods). Velocity was in the upper range of human athletic ability to increase the area that could be searched per unit time.

Our virtual foraging game allowed us to examine the behaviors of more versus less efficient human foragers, as gauged by the number of temples found (all players started in the same location with the same energy budget). We also tested for adaptive behavior, i.e. efficient responsiveness to task and environmental constraints, by manipulating the need to return to a home base, i.e. central-place foraging^42^. This constraint is natural and variable in real-world conditions which suggests that humans and other organisms may have evolved to adjust to central-place variability. The foraging game also elicited the coordination of perceptual search with locomotive search in a relatively natural way. While players did not expend actual physical energy to play the game, they had to decide when and where to look or move to locate resources based on a simulation of the main functional constraints that apply in real human foraging.

**Figure 2:**
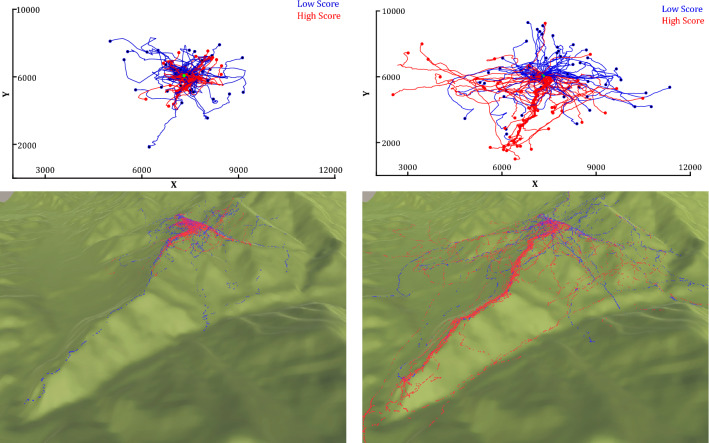
All search trajectories plotted for home-range players (Left) and free-range players (Right), in a 2D plane (Top) and the full 3D terrain (Bottom). Trajectories for low versus high scorers colored separately (blue versus red). The XY coordinates are in meters.

**Figure 3:**
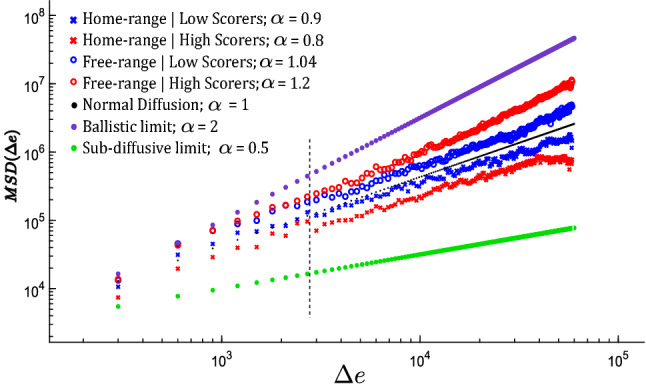
Mean Squared Displacement as a function of energy expended for the ensemble of trajectories in home-range and free-range conditions, separated by low (blue) and high (red) scorers. Functions are also divided into local (left) and non-local (right) scales by the vertical dashed black line, and anchors of normal diffusion and ballistic and sub-diffusive limits are shown for comparison.

Each participant played individually, and each player started on top of a hill where a tower was placed as a home base. The hill was roughly in the middle of a $5\times 5$ km terrain map of the Himalayas that contained 49 temples registered in Google Earth (Fig. 1). We populated the hilly terrain with scattered trees that made temples challenging to see from a distance due to occlusion and misidentification. In the *home-range* condition, players needed to click on the tower to score any temples they acquired up to that point. The players did not need to be next to the home-base to click on it, they could do so from a distance within close vicinity. In the *free-range* condition, temples were scored immediately when acquired, and players did not need to return to the home-base. The game ended only after the energy budget was depleted and the players were awarded 50 cents for finishing the game and an additional 20 cents for each temple scored. Only movements over the landscape cost energy, forward and backward, and players were given an energy budget that enabled them to approach the edges of the game space without reaching them. Energy expenditure was constant for flat and downhill movement and increased linearly with the grade of uphill movement. Given a constant velocity and limited energy budget, the duration of the game was affected by the rate of energy expenditure, for example, more uphill movement increased the rate of energy loss and decreased the total play time.

In typical foraging models, foragers expend energy to move near enough to a resource to perceive it and then move additionally to access it. This additional movement is irrelevant for our purposes, given that the search process is complete at the moment of perceptual detection. To avoid wasting time and energy to walk close enough to physical resources to touch them, players obtained “informational” resources by simply clicking on them upon visual detection.

This informational mode enabled us to measure the perceptual component of foraging, as expressed in reorientations used to scan the landscape for resources visually. It also highlighted the trade-off between visual scanning via reorientations that searched without energy expenditure versus locomotion that brought distant hillsides and other unexplored areas into view. Visual scanning cost time but not energy, whereas locomotion cost both time and energy. Players could increase their play time by taking longer pauses at the cost of time and opportunity. Visual acquisition of resources was reflected in player scores but did not replenish energy levels and the goal was to record as many resources as possible, given a limited energy budget. This goal is based on typical foraging models, where foraging efficiency is defined as maximizing resources as a function of energy integrated over time^43^.

The game was hosted on Amazon’s Mechanical Turk, and 200 unique participants completed the game without technical or other issues–100 in the free-range condition, and 100 in the home-range condition. Players took an average of 12 min to expend their energy budget, and the mean performance for the two conditions was the same at 7.0 temples scored. To assess how the efficiency in foraging behavior interacted with the home-range constraint, we further divided the players based on their scores using a median split, such that the players scoring less and more than the median of 4 were labeled as ‘low-scorers’ and ‘high-scorers’ respectively. We tested how this median split interacted with the manipulation of home base, and we show that bivariate results are consistent with the underlying continuous relationships between measures of foraging and score.

**Figure 4:**
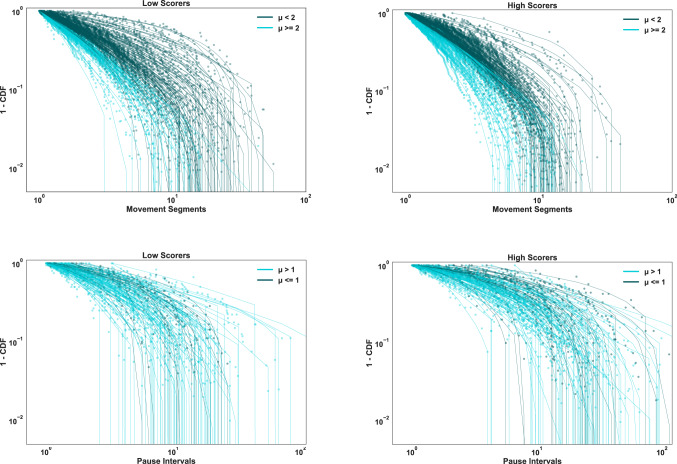
Complementary Cumulative Distribution Function (CCDF or 1 - CDF) for movement segments (**top row**) and pause intervals (**bottom row**) for all low scorers and high scorers. The points represent the empirical data and solid lines show the corresponding truncated power-law fits (using Eq. (2) where $\mu$ is the maximum likelihood estimate for a given empirical distribution, and $x_{min}$ is set to 1 sec). Lines are colored to show the boundaries of $\mu = 2$ for movement segments and $\mu = 1$ for pause intervals.

## Results

### Efficient foraging was more adaptive in response to home-range constraints

All individual search trajectories are shown in Fig. 2 with and without the terrain as the backdrop (top versus bottom) for home-range versus free-range players (left versus right). Before proceeding with analyses, we can observe that foragers generally adapted their movements to task demands in that trajectories were less spread out in the home-range condition. Moreover, this adaptation appears to be more pronounced for high scorers in that the red trajectories appear to be less spread out than blue trajectories in the home-range condition. In contrast, the opposite is true in the free-range condition.

To quantify and test these apparent differences, we used diffusion analysis to measure the degree of spatial dispersion of search trajectories. Diffusion analysis is based on change in mean squared displacement (MSD) over time (*T*), where $MSD \approx T^\alpha$and $\alpha = 1$ corresponds to a random Brownian walk. Subdiffusive ($\alpha < 1$) and superdiffusive ($\alpha > 1$) trajectories indicate anomalous diffusion, where the MSD can either grow slower or faster than expected by a random walk. MSD is usually computed as a function of time, but we computed it as a function of energy expenditure, which is more directly linked with movement dispersal in our game as in real environments.

MSD functions were similar across conditions at local scales (see upcoming segment analyses), but they diverged at longer scales. We calculated MSD from an ensemble average (see Methods) for each condition over all scales to estimate the degrees to which players ranged farther or less far compared with the normal diffusion baseline of $\alpha = 1$ (Fig. 3). We simulated a random walk to confirm that the effect of varying slopes on energy expenditure did not cause a bias away from the baseline of regular diffusion.

**Figure 5:**
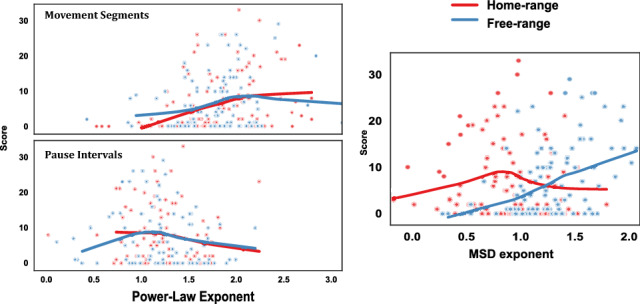
Individual scores as a function of truncated power-law exponents (left) for movement segments and pause intervals, and diffusion exponents (right) for home-range versus free-range foragers. The solid lines show the respective moving averages.

**Figure 6:**
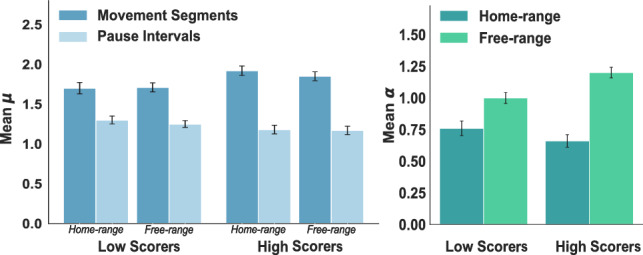
Mean truncated power-law exponents $\mu$ for movement segments and pause intervals (left), and mean diffusion exponents $\alpha$ (right) for home-range versus free-range foragers. Both measures shown separately for low versus high scorers, and error bars show standard errors of the means.

We also calculated MSD for every trajectory separately (Fig. 5) and found that players were significantly superdiffusive in the free-range condition (${\overline{\alpha }} = 1.3$, one-sample *t-test*: $t= 7.8, \, df=99, \,p < 0.001$), and significantly subdiffusive in the home-range condition (${\overline{\alpha }} = 0.86$, one-sample *t-test*: $t= -3.1, \,df=99, \, p = 0.002$). Moreover, the effect of home-range was exaggerated for high versus low scorers as evidenced by a significant interaction between home-range condition and high/low scorers, ${F(1,196) = 11.5,\, p < 0.001}$. We calculated F statistics using two-way (home condition x search efficiency) ANOVA and diffusion exponent estimates $\alpha$ for each player as the dependent variable. Figure 6 shows the mean $\alpha$ values for each condition separated by low versus high scorers. The MSD results confirm that players adapted their overall search trajectories to range farther in the free-range condition, and this adaptation was greater for high scorers.

The home-base served as a resource akin to a temple, i.e., a structure to be located. However, unlike temples, it was a non-destructive resource that players needed to return to, often repeatedly as players would go out on multiple excursions. The memory of the home-base’s location presumably decays over time and distance^44^, so it is beneficial to restrain the dispersal distance when return trips are sufficiently valuable^45^. Efficient search in the home-range condition meant staying closer to the home base, which conserved energy and lessened the burden on memory by keeping the home base tower in view or not far from sight.

### Efficient foraging followed Lévy walks regardless of home-range constraints

The diffusion analyses show how movement trajectories are distributed over long spatial and temporal scales to fit task constraints. Movement trajectories can also be analyzed segment by segment to examine the distribution of movement segments and the degree to which search activity is intensive versus extensive, i.e., relatively short versus long movement segments. The distributions of movement displacements play a role in the use of space and overall diffusion of foraging trajectories, but they can also reveal different patterns at a smaller scale that provide additional information about foraging processes^6^.

We divided foraging trajectories into movement segments and pause intervals, where the former were defined as continuous intervals of forward or backward movement delineated by pauses in movement of any length. Pause intervals were continuous periods of standing still and turning without locomotion. We analyzed the frequency distributions of both kinds of segment distributions by fitting the parameters of several candidate models (see Methods for more details) using maximum-likelihood estimation (*MLE*). The best-fitting model was determined based on relative likelihoods using Akaike Information Criteria (*AIC*) and the Kolmogorov-Smirnov *D* goodness-of-fit metric. The candidate models tested were those most commonly examined in prior foraging studies, and each model was tested against the distribution of each participant.

AIC results showed the large majority of movement segment distributions, $81\%$, were best fit by the truncated power-law model. Therefore, we analyzed the best-fitting parameters of the truncated power-law (Eqn 2) for all trajectories to quantify and compare distributions across conditions (Fig. 4). Estimated power-law exponents were almost entirely within the range of Lévy walks, $1 < \mu \le 3$, and generally close to the theoretical optimum of two, as many studies have found^4,46,47^. $\mu \approx 2$ reflects a balance where shorter, energetically cheaper movements intensively search areas that are reached by longer, energetically more costly movements. The exponent also generates a broad range of step distributions that could help in a judicious sampling of the environment, especially under movement costs and uncertain conditions irrespective of how much space is covered^8,48^. The relationship between exponent and score (Fig. 5) shows that foraging was generally most efficient near $\mu \approx 2$.

In line with Fig. 5, we found that the mean estimated exponents (Fig. 6)for high scorers (${\overline{\mu }} = 1.89$) were reliably closer to optimal compared with low scorers (${\overline{\mu }}= 1.70$), ${F(1,196) = 12.03, \,p = 0.05}$, without any no reliable effect of home-range, ${F(1,196) = 0.15, \,p = 0.7}$. Therefore, while the trajectory diffusion adapted to the home-range/free-range manipulation, the distribution of efficient movement segments resembled a Lévy walk regardless of task constraints. It is especially noteworthy that even the subdiffusive home-range trajectories were composed of Lévy-distributed movement segments. Lévy walks are mostly associated with superdiffusive trajectories and random heading directions that help avoid backtracking and reach unexplored areas. Home-range players were restricted to areas near the home base, but by not picking their headings at random, they were able to maximize their search efficiency within the limited space. Altogether, our results add to previous evidence for the prevalence of $\mu \approx 2$, even when the superdiffusive benefit of Lévy walks is nullified.

### Efficient foraging benefited from concentrated bouts of perception and planning

Foraging in humans and other complex organisms involves perception, memory, planning, and decision-making processes that can be difficult to perform while moving^13^, especially under challenging and uncertain conditions. Pauses between movements are sometimes attributed as “handling times” to retrieve and process resources in optimal foraging literature^17^. In our experiment, pauses between movements were times when players, at the expense of time and effort, could plan their next moves or visually scan the environment for distant resources that may be difficult to discriminate from trees and other environmental features.

**Figure 7:**
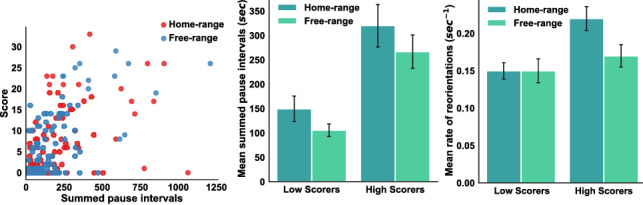
Left: Summed pause intervals plotted against score and separated by home-range versus free-range foraging. Center: Means of summed pause intervals, and Right: mean rates of reorientations. Error bars show standard error of the means.

We first tested whether foraging-related activities were indeed engaged during pause intervals as assumed. We did not have access to direct evidence, but this hypothesis predicts that longer pauses should generally result in better performance due to the value of mental processing. Consistent with this prediction, we found moderately strong correlations between scores and summed pause intervals in both conditions, $r_{home-range} = 0.43, \,p < 0.001$ and $r_{free-range} = 0.60, \, p < 0.001$ (see Fig. 7 (left)). We can infer that at least some time spent during pauses was used for visual scanning to detect temples or the home-base.

Intervals between foraging activities have been studied previously in sit-and-wait ambush predators, where waiting times are distributed according to a power-law^49^ analogous to the distribution of movement segments in Lévy walks^50^. Similar to previous studies, power-law analyses of pause intervals showed that the majority of pause distributions, 76%, were best fit by one of the two power-law models, with 57% truncated Pareto and 19% pure Pareto. We found that $\mu$ parameter estimates for the truncated power-law model were closer to one than two (Fig. 5), consistent with previous results showing evidence for power-law waiting time distributions with $\mu \approx 1$. Moreover, scores were generally highest when estimated exponents for pause intervals were close to $\mu \approx 1$, and exponents for high scorers were closer to one than for low scorers, ${F(1,179) = 3.8, \,p < 0.05}$ (see Fig. 6).

To understand why efficient pause intervals approached $\mu = 1$, we can compare this result with the corresponding analysis of movement segments where the exponent approached $\mu = 2$, which balances energetically costly longer movements with shorter, intensive clusters. For pauses, there is no such energetic cost^51^. To the contrary, relatively long, continuous pauses help to integrate visual information as the environment is scanned, integrate that information with prior knowledge, and consider possible plans for next foraging movements. Such mental processing requires sustained concentration that could be disrupted by too much task switching between movements and pause intervals, or too much difficulty in integrating information across the different viewpoints separated by movement segments. This explanation may be related to previous work showing that $\mu \approx 1$ can result from memory effects on task execution, such that humans perform an activity based on their past activity rate^52^. Better players may be more aware of their activity rates and concentrate their mental efforts accordingly.

### Efficient foraging traded more perception and planning for less exploration with the home-range constraint

We designed our foraging game with an explicit, empirically-based cost to foraging movements, but time and mental effort were inherent costs that players presumably took into account. They could favor exploratory movements over perception and planning, thereby expending energy in exchange for less time and effort, or they could invest more time and effort on careful visual scanning to detect trees or path planning to increase their search efficiency. The home-range constraint favored the latter because of the added energy costs of return trips (although players only needed to see and click the home base to score temples found, some return movement was often needed to bring it into view). The cost of these return trips is reflected in the diffusion results that showed more restricted, subdiffusive search areas with the home-range constraint. If one only takes movement-based search into account, then the need to return to home base should hinder performance because return movements expend the energy budget and thereby reduce the total amount of ground that can be covered.

On the contrary, as reported earlier, mean scores were nearly identical for home-range and free-range foraging. This surprising equivalency implies that home-range players could find almost as many temples as their counterparts without engaging in superdiffusive search. This result indicates that efficient foragers switched their foraging strategies in response to task constraints: Home-range players minimized ranging farther to avoid the movement costs of return trips, but they maintained performance by investing more time in planning or visual scanning. Analogously, optimal foraging models predict that the time spent by foragers in a patch should depend upon travel costs, among other factors^53^. Greater travel costs should increase the probability of exploiting a current location rather than exploring others. We predict that more significant travel costs in the home-range condition should lead players to exploit the area near the home-base by spending more time on planning and visual detection compared with free-range players^54^.

In support of the predictions outlined above (see Fig. 7 center), total time spent on visual processing was 52.47 s longer on average in the home-range versus free-range condition (one-tailed t-test: $t = 1.52, \,df = 198,\, p = 0.06$), and this effect was not reliably different for low versus high scorers. The extra pause time was apparently used for visual search/mental processing helpful to foraging efficiency, as evidenced by the correlation with scores reported earlier. To further corroborate that this extra time spent between movements in the home-range condition was used for visual search, we can find indirect evidence in the frequency and rate of reorientations (turns and head tilts while standing still) that are indicative of active visual scanning and effort (see Supplementary Fig. s1).

As predicted, we found that the rate of reorientation was higher in the home-range condition (see Fig. 7 (right)), $M = 0.18 \,sec^{-1}$, than in the free-range condition, $M = 0.15\, sec^{-1}$ (one-tailed t-test: $t = 1.77,\, df = 198,\, p = 0.035$). Furthermore, high scorers ($M = 0.19 \,sec^{-1}$) reoriented at a higher rate than low scorers ($t= 2.83,\,df=198,\, p = 0.0025$, and high scorers adapted their reorientation rates somewhat more strongly to the home-range manipulation, ${F(1,196) = 2.81,\, p = 0.09}$. These results indicate that the home-range constraint encouraged players to increase their investment in perception and planning, and better players made greater investments than worse players.

## Discussion

In recent years, there has been a tension between physics-based theories and more cognitive theories of foraging^55^. On the one hand, theories like Lévy walks seem plausible for relatively simpler organisms that search for resources relatively uninformed, with little or no memory or directionality, akin to the random walks of particles. These theories mostly focus on optimal movement and assume perfect perceptual capabilities that allow automatic resource detection. On the other hand, cognitive theories that invoke memory, learning, and decision-making seem more appropriate for humans and other complex organisms^56^. Such theories rooted in the optimal foraging framework emphasize trade-offs involving time and energy constraints but devalue the role of spatial search in foraging under constraints on information and memory^38^.

Our results help resolve this tension in the context of a virtual foraging game with realistic human foraging conditions. Foraging behavior looked like an undirected Lévy walk at the level of individual movement segments, but like a directed series of movements and pauses at the level of whole trajectories. Movement segments were unaffected by task constraints and generally more efficient as their power-law exponents approached $\mu = 2$ and pause intervals approached $\mu = 1$. However, movements were clearly not generated by a purely random process when viewed as whole trajectories. Their dispersal respected constraints of the environment and task conditions, and diffusion exponents deviated purposefully from a random walk. To illustrate, the hilly terrain in our virtual Himalayas afforded constraints and opportunities for foraging. As can be seen in Fig. 2, players tended to move along ridges rather than go down to lower elevations, especially in the home-range condition. These trajectories respected constraints of movement costs as well as opportunities for unobstructed visual search.

Lévy walks are generally indicative of superdiffusive search, but we found Lévy distributions in the movement segments of subdiffusive trajectories with the home-range constraint, as well as superdiffusive trajectories without it. Alternatively, players could have altered the diffusivity of search by changing the distributions of movement segments and deviating from a Lévy distribution to a Gaussian distribution with short or long step lengths. However, this method runs the risk of losing scale-invariant search properties of Lévy walks that are beneficial for sampling a space at a large range of scales, especially in uncertain and heterogeneous environments^57,58^. Our results indicate that players controlled movement directions, but not magnitudes, in a way that adapted Lévy-distributed movements to the relevant range of search scales^8,48^ given task constraints. The use of directionality also created movement trajectories that varied in their degree of resampling prior locations, unlike a random Lévy walk. Instead, movements were directed to efficiently over-sample the home-base location at the level of trajectories and thereby enact central-place foraging. We did not test how movement might adapt to variations in the distributions of resource locations, but previous studies have found changes in movement segment distributions with respect to patchiness^8,59^. We only used known temples as resource locations for purposes of ecological validity. However, it would be interesting to examine the effects of different resource distributions and energy landscapes on movement segments versus movement directions in a future study.

We found that pauses for perception and planning were also Lévy-distributed regardless of task conditions^49^, and estimated parameters indicated that efficient foraging benefited from concentrated search effort, as supported by positive correlations between scores and time spent between movements. One role of mental processing was demonstrated in the shift to more visual search in place of less exploration in the home-range condition due to the energetic costs of return trips. Although pauses did not cost energy in the game, they did incur time, effort, and opportunity costs, e.g., spending the time earning money in another task on the MTurk platform^60,61^. In future research, it would be informative to test different time costs and their effects on pauses.

The interleaving of movements with pauses for perception and planning is analogous to inter-patch versus intra-patch foraging, respectively^3,36^—movements take foragers to each new patch i.e. vantage point, and visual search exploits the patch until the probability of finding a resource is low enough to plan and execute the next movement. There is a trade-off between the time and energy needed to move along with the opportunity to find new resources, versus the time needed for visual search and planning to fully cover the area. We found that, in the face of more considerable movement costs, home-range players increased the time they spent on exploiting a ‘patch’. In general, more efficient foraging balanced these trade-offs to create movement trajectories that were judiciously extended or contracted by way of concentrated bouts of visual scanning, orienting, and planning. Less efficient foraging used relatively shorter pauses and longer movement segments arranged in more random configurations, as evidenced by diffusion exponents closer to a random walk. Our home-range constraint was perceptual and hence not energetic up to a certain distance, but more energetic constraints might elucidate the role of memory and decision-making processes that assess probabilistic costs and benefits of farther excursions while saving energy to return home^62,63^.

Our results advocate for integrating physics-based foraging theories with more cognitive approaches to explain how cognition builds on more basic search processes to guide foraging behaviors in humans and other complex organisms. For instance, one approach might posit that Lévy-distributed movement segments serve as building blocks for more cognitively-motivated activities to assemble into movement trajectories. Activities like resource detection, planning, visual scanning would unfold incrementally in bursts of concentrated processing between movements. Previous theories have explained pause intervals in terms of simple models assuming the probability of local target detection increases with longer waiting times^64^. By contrast, our results call for explanations that involve mental processing^20^ and optimal foraging predictions^36,65^. For instance, concentrated bursts of perception and planning might aid attentional focus^66^ similar to area-restricted search. More generally, there is a need to explain how task demands and constraints affect decision processes that span spatial and temporal scales in which local choices over short timescales integrate and interact with broader, longer-range orienting and planning^67–69^. Models of efficient foraging in natural environments should account for the trade-offs between extrinsic constraints (e.g., energy landscapes, resource distributions, landscape heterogeneity) and cognitive constraints (e.g., limited memory and attention, task switching costs, information uncertainty).

In closing, it has been hypothesized that human intelligence partly evolved from adaptations selected for efficient foraging behavior^1,70^. For example, selective attention may have resulted from the tendency of food and other foraging resources to be clustered in time and space, thereby requiring focus on those clusters and not other potentially distracting features in the environment^71^. Likewise, perception and planning decisions may have been shaped by adapting Lévy walks in purposeful ways that adjust to varying conditions and constraints. Such an adaptation may have been co-opted for processes of memory search^33^ and visual search^72^. Understanding the behavioral bases of foraging at different scales^73^ may serve as a foundation for studying trade-offs between physical and cognitive costs involved in many aspects of learning and decision-making^74,75^, with implications for broader problems of optimal search and foraging^48,76,77^.

## Materials and methods

### Game environment

The foraging game was implemented in Unity 3D and primarily scripted in C#. The game was modeled on a $5\times 5$ km area in the Himalayas (Top-Left - 32.6548, 76.056530, Bottom-Right - 32.54895, 76.194889). The relief of the terrain was downloaded from Google Earth and rendered in the Unity environment using Infinity Code. Forty-nine temples were identified in the chosen area based on location data from Google Maps. The coordinates of the temples were marked in the Unity landscape, and a model temple was placed at each location. The movement was only forward and backward with turns to change direction, and movement speed was set to be 6m/s. This fast but realistic speed helped foragers cover a greater area and find more temples in a shorter time that is ideal for MTurk studies. A visible energy bar was depleted as a function of the slope approximated from prior studies on movement associated energy expenditure in humans^41^. Specifically, a constant minimum was set per meter for flat and downhill surfaces, and the cost increased by 35% for each angular degree of increase in grade. This approximation is a simplification of more realistic functions that consider higher-order effects of downhill slopes on energy expenditure. We also simplified the game by excluding a resting metabolic cost during pauses and turns, and excluding an energetic reward for finding temples. These simplifications made it easy to measure and compare foraging efficiencies while also controlling the length of game play.

Players used six keys to control movement and perspective (‘w,’ ‘a,’ ‘s,’ ‘d,’ ‘up arrow,’ ‘down arrow’), the latter two tilting their line of sight up and down to adjust for sloping terrain. Mouse movements were used for clicking on temples in view when found, and auditory feedback was available with steps to indicate movement, and clicks to indicate successful temple identification. Players were recruited on Amazon Mechanical Turk and the study was approved by the University of California Merced Institutional Review Board. All experiments and analyses were performed in accordance with the guidelines of the review board. Informed consent was obtained and players were given a guided practice trial to acclimate to the game, and its rules and controls. They were instructed to find as many temples as possible before depleting their energy, with the number of temples displayed in the corner of the screen. In the home-range condition, where players clicked on the home-base to report temples, the number of temples found and reported back to base were both displayed. Player scores were based only on the number of temples reported, and to calibrate, players were told that 49 temples existed in the game. Each player was paid a base amount of 50 cents for completing the game, and an additional bonus of 20 cents was awarded for every temple successfully recorded in free-range condition. However, for the home-range condition, the bonus was awarded for every temple reported back to the home-base. Game can be accessed at Home-range and Free-range.

### Trajectory analysis

We computed MSD as a function of energy expended to characterize the type of diffusion:

1 $$\begin{aligned} MSD = \frac{1}{n} \sum _{j=1}^{n} <[r(e + \Delta e) - r(e)]^2>_j \end{aligned}$$

where *r(e)* is the position vector of the player at energy *e*, $\Delta$ *e* is the energy expended, *n* is the total number of players.

### Segment distribution analysis and model selection

The game play for each participant was divided into segments of continuous movement (i.e. the forward or backward key was held down) interspersed with time intervals of no movement, i.e. pause intervals. The length of each movement segment *l* was the total amount of time spent moving during the segment, which was equivalent to the amount of ground covered because velocity was constant. We discarded segments where *l* was less than one second.

Distributions of individual movement segments or pause intervals were plotted and analyzed directly, rather than binned into frequency histograms^78^. We calculated complementary cumulative distribution functions (i.e. CCDF or 1-CDF) for a given distribution function *X*, evaluated at every observed segment length, *l*, where CCDF(*l*) = $P(X \ge l)$ or the probability of observing segments in a given distribution $\ge$ *l*. CCDFs were plotted simply by rank ordering individual segments along the x-axis, with CCDF(*l*) on the y-axis. We calculated and plotted individual CCDF for each participant instead of combining them into a single distribution.

We used Maximum Likelihood Estimation (MLE) to fit each distribution to the probability density functions of the most commonly used models in behavioral and foraging studies: Truncated power-Law, Pareto, lognormal, truncated Pareto with an exponential cutoff, exponential, and bi-exponential distributions. MLE finds the PDF parameters that maximize a monotonic log-likelihood function ${\mathcal {L}}(\mu )$ such that ${\mathcal {L}}(\mu _{max}|x) > {\mathcal {L}}(\mu _{other}|x)$ where *x* is the data and $\mu$ is the fitted parameter.

We estimated the goodness-of-fit between data and model by calculating the log-likelihood values for estimated model parameters, $L = \sum _{i=1}^{n} {\mathcal {L}}(\mu _{max}|x_{i})$, i.e. summation of the likelihood function ${\mathcal {L}}$ over all *n* data points in the distribution. We then selected the best model using the Akaike Information Criteria (AIC) which prefers the model with fewest parameters and maximum *L*. We used the small-sample correction for the AIC when needed. We also calculated Kolmogorov-Smirnov (KS) *D* statistic between CCDFs of empirical data and model estimates to further check the goodness-of-fit. The KS statistic measures the maximum distance between two distributions, KS results were in agreement with AIC results.

Model parameters and estimates are available in the supplementary datasets. Truncated power-law, bi-exponential and Pareto were the best fitting probability density functions (parameters were estimated numerically):

Truncated power-law:

2 $$\begin{aligned} p(x) = \frac{\mu \left( x_{max}^\mu - x_{min}^\mu \right) }{x_i^{\mu +1}} \end{aligned}$$

where parameters estimated numerically were $\mu$ and $x_{max}$. Power-law:

3 $$\begin{aligned} p(x) = \frac{\mu \left( x_{min}^\mu \right) }{x_i^{\mu +1}} \end{aligned}$$

where $\mu$ was estimated numerically. Bi-exponential:

4 $$\begin{aligned} p(x) = A exp(-\lambda _{1} x_i) + (1-A) exp(-\lambda _{2} x_i) \end{aligned}$$

where *A* and $1-A$ are the relative weights of the two modes, and ${\lambda _{1}}$ and $\lambda _{2}$ are the exponential decay rates.

The bi-exponential model provided good fits to the data, but it was not favored by AIC because of its additional parameter. AIC results showed that a large majority of distributions were best fit by the truncated power-law model. Therefore, as shown in Fig. 4, we plotted CCDFs of each individual distribution and its best-fitted truncated power-law function.

## Supplementary information

Supplementary material 1 (pdf 67 KB)

Supplementary material 2 (csv 11 KB)

Supplementary material 3 (csv 12 KB)

Supplementary material 4 (csv 12 KB)

Supplementary material 5 (csv 11 KB)

Supplementary material 6 (csv 12 KB)

Supplementary material 7 (csv 12 KB)

Supplementary material 8 (csv 13 KB)

Supplementary material 9 (csv 12 KB)

Supplementary material 10 (csv 18 KB)

Supplementary material 11
