# Supplementary material for: Efficient Lévy walks in virtual human foraging
Source: Sci Rep. 2021 Mar 4;11:5242. doi: 10.1038/s41598-021-84542-w (PMC7933158; doi:10.1038/s41598-021-84542-w)
Supplement: Supplementary file 1 — Supplementary material 1 (pdf 67 KB) [file 41598_2021_84542_MOESM1_ESM.pdf]

Supplementary Figures

Ketika Garg, Christopher Kello

January 2021

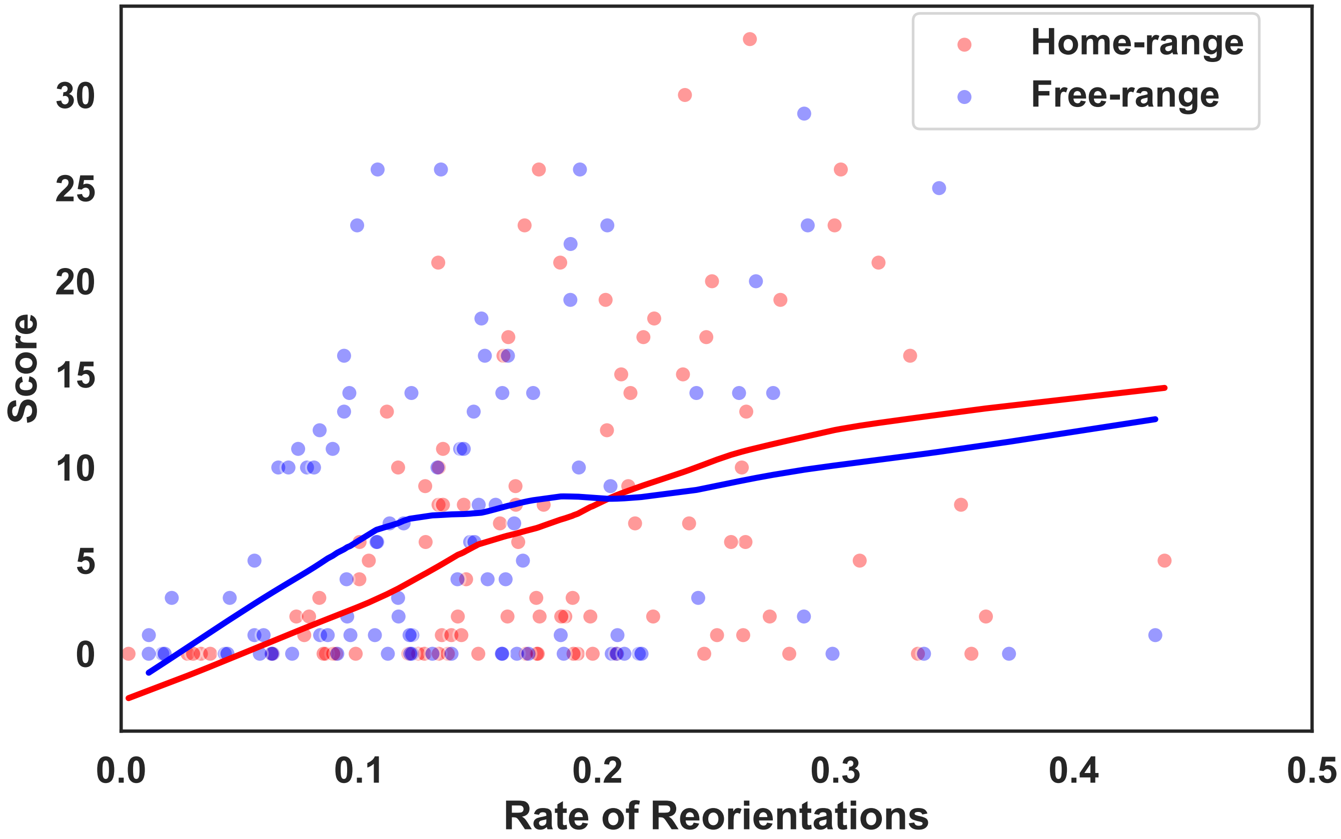

Supplementary Figure 1: Individual scores as a function of rate of reorientations for home-range versus free-range foragers. The solid lines show the respective moving averages.

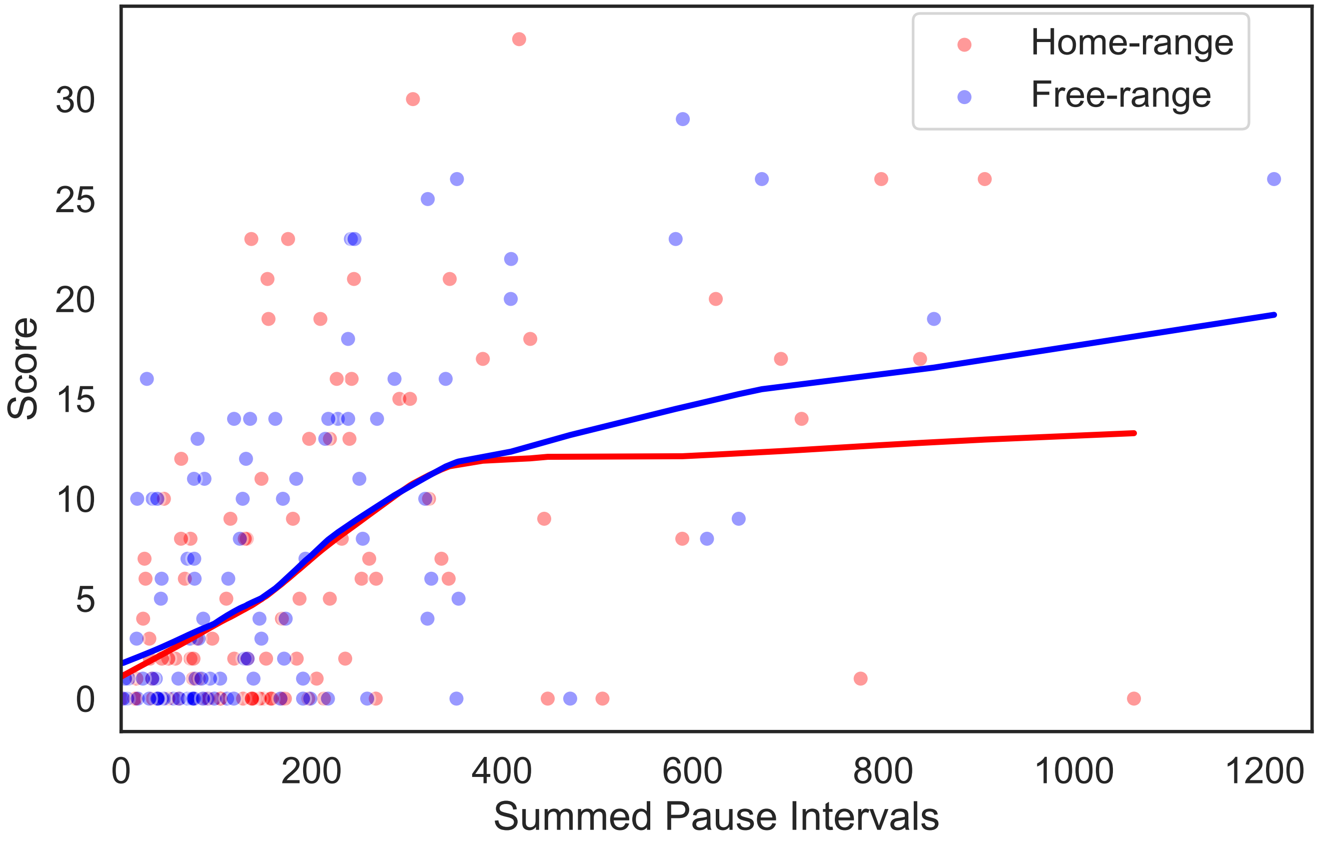

Supplementary Figure 2: Individual scores as a function of total duration of pauses for home-range versus free-range foragers. The solid lines show the respective moving averages.
