# Supplementary material for: Efficient Lévy walks in virtual human foraging
Source: Sci Rep. 2021 Mar 4;11:5242. doi: 10.1038/s41598-021-84542-w (PMC7933158; doi:10.1038/s41598-021-84542-w)
Supplement: Supplementary file 11 — Supplementary material 11 [file 41598_2021_84542_MOESM11_ESM.pdf]

##readme

##23_Apr_2020

##KetikaGarg

- Supplementary file 1 : this file contains the key parameters from the results of each individual player.
- The following files contain parameters from model selection on the distribution of movement intervals for every player divided by their score and condition:
    - Supplementary file 6: High scorers and free-range
    - Supplementary file 7: High scorers and home-range
    - Supplementary file 8: Low scorers and free-range
    - Supplementary file 9: Low scorers and free-range

- The following files contain parameters from model selection on the distribution of pause intervals for every player divided by their score and condition:
    - Supplementary file 2: High scorers and free-range
    - Supplementary file 3: High scorers and home-range
    - Supplementary file 4: Low scorers and free-range
    - Supplementary file 5: Low scorers and free-range

**Model selection:**

- Using MLE, parameters for lognormal(ln), exponential(exp), power-law(pl), truncated-powerlaw(tpl), bi-exponential(biex) were estimated.
- 
- AIC weights were calculated for each of the model (in the data files, AIC values for each model are under column 'aic_{model}' and AIC weights are referenced by 'aicw_{model}').
- Goodness-of-fit test was done using Kolmogorov-Smirnov statistic (in the data files, D-statistic for each model is referenced by 'ksd_{model}' and the associated p-values are under 'ksp_{model}'.

- Example: key statistics for lognormal models are under columns 'aic_ln', 'aicw_ln', 'ksd_ln', 'ksp_ln'.

- Majority of distributions were fit by both Truncated-powerlaw and Biexponential models, thus truncated power-law exponent (mu) and bi-exponential parameters (weight - a1 and decay parameters - l1,l2) are provided for every player.
